# Immunosuppressive therapy in patients with biopsy-proven inflammatory myocardial disease: a systematic review and meta-analysis

**DOI:** 10.1038/s41598-025-25165-3

**Published:** 2025-10-23

**Authors:** Bruno Stautner, Michał Tkaczyszyn, Anne Sorg, Tomasz Suchocki, Ewa Anita Jankowska

**Affiliations:** 1 Deutsches Herzzentrum Der Charité, Universitätsmedizin Berlin, Augustenburger Pl. 1, 13353 Berlin, Germany; 2 Division of Translational Cardiology and Clinical Registries, Institute of Heart Diseases, Wroclaw Medical University, Wroclaw, Poland; 3 Institue of Heart Diseases, Jan Mikulicz Radecki University Hospital in Wrocław, Wroclaw, Poland; 4 Faculty of Medicine, Wroclaw Medical University, Wroclaw, Poland; 5 Department of Genetics, Theta - the Biostatistics Group, Wrocław University of Environmental and Life Sciences, Wrocław, Poland

**Keywords:** Heart failure, Myocarditis, Cardiomyopathy, Immunosuppression, Meta-analysis, Systematic review, Cardiology, Heart failure, Immunosuppression

## Abstract

The aim of this study was to evaluate and update the effects of immunosuppressive therapy on heart failure outcomes in patients aged 16 years or older with biopsy-proven inflammatory myocardial disease. We performed a systematic review and an aggregate data meta-analysis of studies evaluating the effects of such therapy in acute and chronic myocarditis or inflammatory dilated cardiomyopathy. Studies were chosen based on criteria related to clinical relevance for therapeutic decisions and measured outcomes in left ventricular ejection fraction (LVEF) and New York Heart Association classification (NYHA) at 3, 6, and 12 months. Data sources included PubMed, Embase, Ovid, and ClinicalTrials.gov, with manual reference checks. Data extraction and risk-of-bias assessments were performed by two independent reviewers. We used a random-effects model and assessed heterogeneity with I^2^ and τ^2^ statistics. . Seven eligible studies with a total of 594 patients were included, that investigated clinical course of acute and chronic myocarditis and inflammatory dilated cardiomyopathy. At 3 months, the pooled standardized mean difference (SMD) for LVEF was 0.97 (95% CI –0.05 to 1.99; *p* > 0.05). At 6 months, the pooled SMD was 0.48 (95% CI –1.31 to 2.28), and at 12 months, –0.01 (95% CI –1.65 to 1.62). For NYHA classification, pooled SMDs were 0.02 (95% CI –0.21 to 0.25) at 3 months and –0.22 (95% CI –1.56 to 1.11) at 12 months. In all cases, confidence intervals crossed zero. Heterogeneity remained high (I^2^ = 92–97%), reflecting substantial variability across studies. In patients with acute or chronic myocarditis or inflammatory dilated cardiomyopathy, immunosuppressive therapy does not improve key echocardiographic or clinical parameters when adding new data from randomized controlled trials (RCTs). High heterogeneity suggests variability in patient populations and protocols, highlighting the need for well-designed RCTs. There is still no evidence to support routine endomyocardial biopsy in non-severe disease, as it will not impact symptomatic treatment, even if inflammation is present.

**Supplementary Information:**

The online version contains supplementary material available at 10.1038/s41598-025-25165-3.

## Introduction

Myocarditis and inflammatory dilated cardiomyopathy are characterized by inflammation of the myocardium, which can occur with or without the presence of viral pathogens and can disrupt cardiac function, leading to a spectrum of clinical manifestations ranging from mild chest pain to severe heart failure^1^. Definitive diagnosis is often achieved through endomyocardial biopsy (EMB), which provides histological evidence of inflammation^2–4^.

Current knowledge indicates that a mild form of the disease often resolves on its own^5^, while up to 30% of biopsy-confirmed cases progress to dilated cardiomyopathy (DCM) with a poor prognosis^2–8^. The etiology of myocardial inflammation serves as a prognostic indicator for the disease’s outcome and guides specific therapeutic approaches^5^.

Despite ongoing research, there remains no clear evidence supporting interventions beyond observation and supportive care for most cases of inflammatory myocardial disease, except in specific conditions that require immunosuppressive therapy, such as giant-cell myocarditis, cardiac sarcoidosis and eosinophilic myocarditis.^9,10^. It is important to note that this applies to certain forms of myocardial inflammatory disease, where the need for immunosuppression is well-established, and not to other types where the role of immunotherapy remains uncertain.

Our systematic review and meta-analysis examines randomized controlled trials (RCTs), including studies that have not been previously evaluated, and aims to assess whether immunosuppressive therapy, either as monotherapy or in combination, provides benefits for patients with endomyocardial biopsy-proven myocardial inflammation and heart failure-associated burden, based on clinical and echocardiographic parameters that guide the initiation of immunosuppressive therapy and serve as follow-up measures in the adult population. Previous trials and meta-analyses have yielded conflicting results due to heterogeneity in diagnostic criteria and inclusion of non-biopsy-confirmed cases, limiting reliability. To address this, our systematic review and meta-analysis includes only randomized controlled trials enrolling patients with biopsy-proven myocardial inflammation. By examining these heart failure-related outcomes, we aim to clarify whether immunosuppressive therapy could offer valuable therapeutic options for patients with diverse forms of myocardial inflammatory disease and associated heart failure in the future.

## Methods

We performed a systematic review and an aggregate-data meta-analysis according to the established standards of the Preferred Reporting Items for Systematic Reviews and Meta-Analyses (PRISMA) guidelines for interventional studies^11^ and diligently followed its comprehensive checklist.

### Eligibility criteria

The inclusion criteria for this meta-analysis and systematic review were established by the three principal authors (B.S., A.S., M.T.). Studies were required to include patients, 16 years or older, diagnosed with acute, chronic, or subacute manifestations of myocardial inflammation associated with heart failure, confirmed by endomyocardial biopsy. The intervention involved treatment with immunosuppressive therapy as part of standard heart failure management. The comparison group had to receive either a placebo or an alternative pharmacological regimen. Due to its clinical relevance, the outcomes included are LVEF and/or the NYHA classification to assess the effect of empirical treatment with medications that prevent cardiac remodeling (anti-remodeling therapy). Additionally, only RCTs, either two-arm or multi-arm designs, were included. The language of publication was restricted to English.

### Information sources

The authors, B.S and A.S, systematically and independently of each other conducted a search on PUBMED, EMBASE, OVID, and ClinicalTrials.gov. Cochrane CENTRAL and Scopus were not included in the search strategy due to substantial overlap with the databases already selected. Cochrane CENTRAL primarily indexes trials also found in MEDLINE and EMBASE, both of which were directly searched. Similarly, Scopus, while broad in scope, was not expected to provide additional relevant clinical trial data beyond what is available through PUBMED, EMBASE, and OVID. Therefore, the selected databases were considered sufficient to ensure a comprehensive and methodologically coverage of the available evidence. This search encompassed studies from January 1989 to June 2025. The authors utilized keywords and Medical Subject Heading (MeSH) terms related to myocarditis, cardiomyopathy, types of therapy, individual drug names, and drug classes. To narrow down the results, clinical trials and randomized controlled trials were specifically filtered for. Reference lists of publications were screened for further articles that met our inclusion criteria to identify articles not found in the predefined automated search. Additionally, study authors were contacted directly in case of incompleteness of their presented study materials. Unpublished works or conference proceedings were not included.

### Study selection

We identified all randomized controlled trials investigating the outcome of immunosuppressive therapy to treat patients with biopsy-proven myocardial inflammatory disease. The two authors B.S and A.S screened titles and abstracts and checked for the availability of full text versions including the study rationale and design. Studies that are covered within this meta-analysis and systematic review included patients with acute, chronic and subacute forms of diverse myocardial inflammation excluding other types such as giant-cell, eosinophilic, checkpoint inhibitor induced, fulminant, rheumatic and Kawasaki forms of the disease due to its heterogenous presentation and pathophysiological differences in its presentation and treatment options. Remaining articles were examined for inclusion based on previously mentioned criteria with comprehensive assessment of their full.

### Protocol and registration

This review was not registered in PROSPERO due to the retrospective nature of the analysis, limited time to complete registration before data extraction, and the fact that the review evolved from a broader project whose scope was refined after data collection had begun.

### Data collection process

Data were independently extracted by the two authors B.S and A.S using a dedicated data extraction form. When needed, discrepancies were adjudicated by the third author—M.T.

We gathered information on various aspects, including patient baseline characteristics, a description of the patient population, trial design, major inclusion criteria, participating centers, detailed treatment information such as information about the comparator, drugs used, their dosages, follow-up duration, and endpoints related to LVEF% and NYHA, if available.

For additional details, we conducted searches to access the study rationale and design and directly contacted study authors.

### Risk of bias in individual studies

Risk of bias were assessed at a study and outcome level using dedicated tool for randomized studies, Cochrane RoB 2.0^12^. For this purpose, we evaluated information concerning randomization (1), blinding and deviations from planned interventions (2), availability of data (3) methods of outcome measuring (4) and choice of reported data (5). Risk of Bias were assessed independently by the two authors B.S and A.S. Any discrepancies were further evaluated and judged by M.T.

### Statistical analyses

Continuous variables (LVEF) and ordinal variables (NYHA class) were analyzed by calculating the mean difference (MD) compared with the control group with 95% confidence intervals (95% CI) using R Software^12^ with a standard deviation (SD) of the mean as the measure of the effect of immunosuppressive therapy. The mean change was calculated based on specific time points of 3, 6, and 12 months, respectively, between placebo and treatment group for LVEF. The mean change for NYHA class was calculated based on specific time points of 3 and 12 months, respectively.

In randomized controlled trials that included information about SD, we used a pooled standard deviation. If there was no information in the RCT about SD, we created a 1000 bootstrap samples with other parameters presented in the paper and we used bootstrap estimator of pooled SD.

Using Q-Cochrane and I^2^ statistics, we assessed the heterogeneity between the studies included in this meta-analysis, with a *p*-value of < 0.10 considered statistically significant.

We conducted an aggregate data meta-analysis of randomized controlled trials using a random-effects model. For binary outcomes, we employed odds ratios, while for continuous outcomes, we used the standardized mean difference.

We calculated pooled estimates of the standardized mean difference (SMD) using a random-effects model, while a *p*-value < 0.05 was considered statistically significant.

All analyses were performed using R software version 3.0.3^12^ and the STATISTICA 12 data analysis software system (StatSoft Inc.).

## Results

**Fig. 1:**
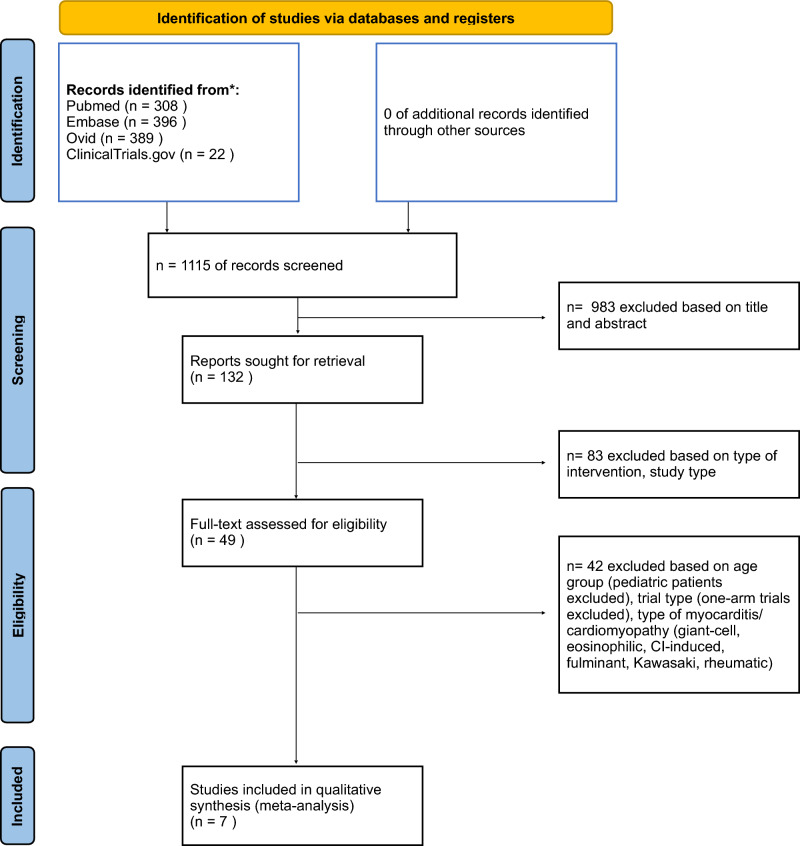
PRISMA flow diagram. The PRISMA flow diagram depicts the flow of information through different phases of this systematic review. It maps out the number of records identified, included and excluded, and the reasons for exclusions.

A total of 1115 records were initially identified and screened. Of these, 983 records were excluded based on their title and abstract. Subsequently, 132 full-text reports were reviewed for eligibility. Among these, 83 reports were excluded due to the type of intervention or study design. Ultimately, 49 records underwent a thorough full-text assessment, and 42 were excluded due to factors such as the focus on specific age groups (e.g. pediatric studies), study design, or type of myocardial inflammation, as outlined in the study selection process. The primary literature research was repeated by the second reviewer (A.S), blinded to the results of the initial study selection, and the same list of seven studies was obtained. In the end, a total of seven studies were included in the quantitative synthesis (meta-analysis). The process of article selection followed the PRISMA guidelines, as illustrated in Fig. 1.

### Characteristics of included studies

Seven eligible randomized controlled trials^13–19^ yielding a total number of 594 patients were finally included in the following analyses. The included studies focused on patients with biopsy-proven myocardial inflammation, specifically acute, chronic, and subacute forms, while excluding certain types of myocarditis. Restricting the population to adolescents and adults (16 years or older) ensured consistency in age-related disease presentation and treatment response. This targeted approach aimed to isolate the effects of immunosuppressive therapy on a uniform cohort without confounding from other myocarditis types or age-related variability. Comparison of studies as well as general characteristics are summarized in Tables 1 and 2.

**Table 1:** Comparison of included studies in this meta-analysis.

	Hazebroek et al.^13^	Wojnicz et al.^15^	Frustaci et al.^16^	Schultheiss et al.^17^	Parrillo et al.^18^	Poloczkova et al.^14^	Mason et al.^19^
Design							
Major inclusion criteria	LVEF < 45%, idiopathic chronic DCM	Chronic HF due to DCM: dyspnea and/or fatigue at rest or exertion, > 6 months, EF ≤ 40%	Dilatation and left ventricular dysfunction (LVEF < 45%), age 18–75, chronic HF (> 6 months) unresponsive to conventional therapy, histologic/immunohistochemical evidence of active lymphocytic MYO, absence of PCR evident cardiotropic viruses, No congenital/valcular/CAD	Chronic viral CMP, Chronic HF symptoms/left ventricular dysfunction > 6 months despite OMT (dyspnea, fatigue, impaired exercise capacity, peripheral edema), age 18–75	LVEF < 35%, Symptoms of CHF due to DCM with exclusion of coronary, hypertensive, valvular, congenital or toxic causes	LV systolic dysfunction (LVEF ≤ 40%), age 18–65, Duration of symptoms; CZECH-ICIT1; 2 weeks – 6 months, CZECH-ICIT2; more than 6 months, HF Symptoms NYHA II-IV with biopsy confirmed myocardial inflammation	Histologic evidence of Myocardial inflammation, LVEF < 45%
Disease	Chronic DCM	Chronic MYO, inflammatory dilated CMP	Inflammatory CMP	Chronic CMP	DCM	Inflammatory CMP	MYO
Presence of inflammation/viral presence	Inflammation + , Virus +	Inflammation + , Virus -	Inflammation + , Virus -	Inflammation + , Virus +	–	Inflammation + , Virus – (low PVB19 load allowed)	Inflammation + , Virus: not evaluated
Intervention	IVIG	Prednisone + Azathioprine	Prednisone + Azathioprine	Interferon- ß-1b	Prednisone	Prednisone + Azathioprine	Azathioprine or Cyclosporine + Prednisone
Intervention dosing rules	IVIG 2 g/kg	*Prednisone* Starting dose: 1 mg/kg/d. Maintenance: 0.2 mg/kg/d for 90 days *Azathioprine* 1 mg/kg/d for 100 days	*Prednisone* 1 mg/kg/d for 4 weeks, followed by 0.33mg/kg/d for 5 months *Azathioprine* 2 mg/kg/d for 6 months	(A): 2 MIU IFN- ß -1b in week 1, 4 MIU IFN- ß -1b week 2 to 24, every other day. (B): 2 MIU IFN- ß -1b in week 1, 4 MIU IFN- ß -1b week 2–3, 8 MIU IFN- ß -1b week 4–24, every other day	Oral Prednisone, 60 mg/d for 3 months	(A) Prednisone (90 d) 1 mg/kg/d for 12 days, every 5 days reduced to 0.2 mg/kg/d, Azathioprine 1 mg/kg/d (100 d) (B) Prednisone 1 mg/kg/d for 4 weeks then 0.33 mg/kg/d for 5 months, Azathioprine 2 mg/kg/d for 6 months	(A) bd 1 mg/kg Azathioprine for 24, Prednisone 1.25 mg/kg/d for 1 week, decreased by 0.08 mg/kg/week until 0.33 mg/kg/d at week 12 to week 20, then tapered off until week 24. (B) bd oral cyclosporine 5 mg/kg to achieve concentration of 200–300 ng/ml at week 1, then tapered off to 100–200 ng/ml at week 2 to 4. 60–150 ng/ml until week 24. Prednisone 1.25 mg/kg/d for 1 week, decreased to 0.15 mg/kg/d at week 23, halved to week 24
Centres	Single-centre	Two-centre	Single-centre	–	–	Multi-centre	Multi-centre
Randomization	Yes	Yes	Yes	Yes	Yes	Yes	Yes
Comparator	Placebo	Placebo	Placebo	Placebo	OMT	OMT	OMT
Blinding	Double-blind	Open-label	Double-blind	Double-blind	Open-label	Open-label	Open-label
Treatment time	4 days	Prednisone 90 days, Azathioprine 100 days	6 months	6 months	3 months	*First arm*: Prednisone for 90 days, Azathioprine for 100 days. *Second arm*: Prednisone + Azathioprine for 6 months	6 months
Follow-up (assessments)	2 weeks, 3 months, 6 months	3 months, 6 months, 12 months, 24 months	6 months	6 months	3 months, 9 months, 15 months	1 month, 3 months, 6 months, 12 months	12 weeks, 28 weeks, 52 weeks
Power analysis	Yes	No	Yes	No	Yes	No	Yes
Endpoints: parameters							
LVEF	+	+	+	–	+	+	+
NYHA	–	+	–	+	+	+	–

Overview table containing information on (1) Design; major inclusion criteria, diseases, viral presence/presence of inflammation, intervention, intervention dosing rules, centres, randomization, comparator, blinding, treatment time, follow-up and (2) Endpoint parameters; LVEF, NYHA. *DCM* dilated cardiomyopathy, *LVEF* left ventricular ejection fraction, *NYHA* New York Heart Association Classification, *IVIG* intravenous immunoglobulin, *HF* heart failure, *MYO* myocarditis, *CMP* cardiomyopathy, *CAD* coronary artery disease, *OMT* optimal medical therapy, *CHF* congestive heart failure, *PVB19* parvovirus B19, *bd* twice daily, *MIU* milli international unit.

**Table 2:** Baseline characteristics.

	Hazebroek et al.^13^			Wojnicz et al.^15^		Frustaci et al.^16^		Schultheiss et al.^17^			Parrillo et al.^18^			Poloczkova et al.^14^		Mason et al.^19^		
	Treatment		Control	Treatment	Control	Treatment	Control	Treatment 1 (4MIU)	Treatment 2 (8MIU)	Control	Treatment	Control		Treatment	Control	Treatment		Control
No. of patients randomized	26		24	41	43	43	42	49	46	47	49	53		9	11	64		47
No. of patients who completed the study	26		24	28	30	43	42	131*			49	52		9	11	NA		
Age (years)	54 ± 13		53 ± 9	41 mean (16,61; 95% CI)	39 mean (29,60; 95% CI)	44.2 ± 15.8	41.1 ± 15.1	47.4 ± 12.3	53.1 ± 10.3	51.4 ± 11.1	43 mean (23,67; 95% CI)			46.1 ± 7.3**		43 ± 14	41 ± 13	
Female sex (%)	23%		21%	21.95%	13.95%	41%	38%	24.5%	39.1%	45.8%	NA			10% **		42.2%	31.9%	
I (NYHA)	NA	NA		0	0	0	0	0	0	0	NA			2.2 ± 0.4***	2.1 ± 0.3***	14.5%	15.6%	
II (NYHA)	NA	NA		8 (19.51%)	15 (34.88%)	22 (51%)	26 (62%)	27 (55%)	28 (61%)	34 (72%)	NA					38.7%	28.9%	
III (NYHA)	1 (4%)		0 (0%)	29 (70.73%)	26 (60.46%)	15 (35%)	12 (29%)	18 (37%)	18 (39%)	13 (28%)	NA					38.7%	42.2%	
IV (NYHA)				4 (9.75%)	2 (4.65%)	6 (14%)	4 (9%)	1 (2%)	0	1 (2%)	NA					8.1%	13.3%	
LVEF (%)	36 ± 7		35 ± 6	23.8 ± 8.6	24.9 ± 7.3	26.5 ± 6.6	27.7 ± 6.4	45 ± 12.4	43.2 ± 10.5	44.1 ± 10.6	17.9 ± 1.0		17.1 ± 1.1	22.3 ± 4.7	21.7 ± 4.0	24 ± 11.0	24 ± 9.0	
LVEDD (mm)	NA		NA	65.6 ± 8.6	68.5 ± 8.0	68.4 ± 7.0	68.9 ± 7.5	57.5 ± 10.7	58.8 ± 7.8	60 ± 9.6	69.8 ± 1.4		67.7 ± 1.0	64.0 ± 8.9	64.8 ± 7.0	NA	NA	
Atrial fibrillation, n (%)	5 (19%)		10 (42%)	11 (26.8%)	14 (32.5%)	NA	NA	8.7%	6.3%	12.2%	NA		NA	NA	NA	NA	NA	
Presence of bundle branch block	5 (19%) (LBBB)		6 (25%) (LBBB)	NA	NA	5 (12%) (LBBB)	8 (19%) (LBBB)	13.0%	12.5%	10.2%	NA		NA	NA	NA	NA	NA	

Overview table containing baseline characteristics of included studies. *DCM* dilated cardiomyopathy, *LVEF* left ventricular ejection fraction, *NYHA* New York Heart Association Classification, *LVEDD* left ventricular end-diastolic diameter, *LBBB* left bundle branch block, *NA* not answered. *12 patients did not complete the study (no information about their arm). **mean age with standard deviation only given for the whole group. ***NYHA class given in mean + standard deviation, no further division.

### Risk of bias in studies

The seven selected studies were independently appraised by the two investigators (B.S, A.S). Discrepancies were carefully reviewed and resolved by the third investigator (M.T). In the studies by Wojnicz^15^, Parrillo^18^, Poloczkova^14^, and Mason^19^, there was no clear information provided about the allocation sequence, leaving uncertainty as to whether it was truly random and whether the allocation was concealed until participants were enrolled and assigned to an intervention. This raises potential concerns about selection bias.

Regarding blinding, three studies employed a double-blind design^13,16,17^, whereas four studies were open-label^14,15,18,19^. The open-label design of these studies increases the risk of performance and detection bias, as participants and assessors were aware of the intervention received. The risk of bias due to missing outcome data was evident in the studies by Parrillo et al.^18^, and Mason et al.^19^. Parrillo et al.^18^ reported outcome data for the majority of participants but did not provide full details on echocardiographic examination data. Similarly, Mason et al.^19^ did not have complete outcome data for all patients at the 28 week and 52 week follow-ups, and to address this, the authors employed last-observation-carried-forward methods using the last available LVEF values for patients who did not complete the study. This could potentially introduce bias into the results.

Furthermore, the risk of bias in the measurement of outcomes arose in three studies^14,15,19^, primarily due to the unblinding of participants to their interventions. This unblinding raises concerns about detection bias, especially for subjective measures such as NYHA classification.

Lastly, there was no clear indication as to whether a pre-specified analysis plan was used in three studies^15,18,19^. The absence of a clearly defined analysis plan introduces the potential risk of selective reporting bias, as post hoc decisions regarding data analysis could have influenced the reported outcomes.

In conclusion, the overall risk of bias is deemed low in Hazebroek et al.^13^, Frustaci et al.^16^, and Schultheiss et al.^17^, while there are some concerns regarding the risk of bias in Wojnicz et al.^15^, Poloczkova et al.^14^, and Mason et al.^19^. Only Parrillo et al.^18^ shows a high overall risk of bias.

The availability of particular variables included in this systematic review and meta-analysis of the seven selected studies is summarized in Table 3. The full assessment of risk of bias using the Cochrane Collaboration’s tool for each study is provided in the Supplementary Data.

**Table 3:** The cochrane collaboration’s tool for assessing risk of bias in randomized trials.

Study	Randomisation process	Deviations from the intended interventions	Missing outcome data	Measurement of the outcome	Selection of the reported result	Overall
Hazebroek et al.^13^	( +)	( +)	( +)	( +)	( +)	( +)
Wojnicz et al.^15^	(!)	( +)	( +)	(!)	(!)	(!)
Frustaci et al.^16^	( +)	( +)	( +)	( +)	( +)	( +)
Schultheiss et al.^17^	( +)	( +)	( +)	( +)	( +)	( +)
Parrillo et al.^18^	(!)	(!)	(–)	( +)	(!)	(–)
Poloczkova et al.^14^	(!)	(!)	( +)	(!)	( +)	(!)
Mason et al.^19^	(!)	( +)	( +)	(!)	(!)	(!)
( +) Low risk of bias; (–) high risk of bias; (!) some concerns						

The risk of bias 2 (RoB 2) tool assesses the risk of bias in randomized trials, assessing (1) Randomization Process, (2) Deviations from the intended interventions, (3) Missing outcome data, (4) Measurement of the outcome, (5) Selection of the reported result, (6) Overall risk of bias.

### Meta-analysis

#### Effects of immunosuppressive therapy related to left ventricular ejection fraction (LVEF)

We assessed the impact of immunosuppressive therapy on LVEF at a 3 month follow-up using data from Hazebroek et al.^13^, Wojnicz et al.^15^, and Parrillo et al.^18^.

**Fig. 2:**
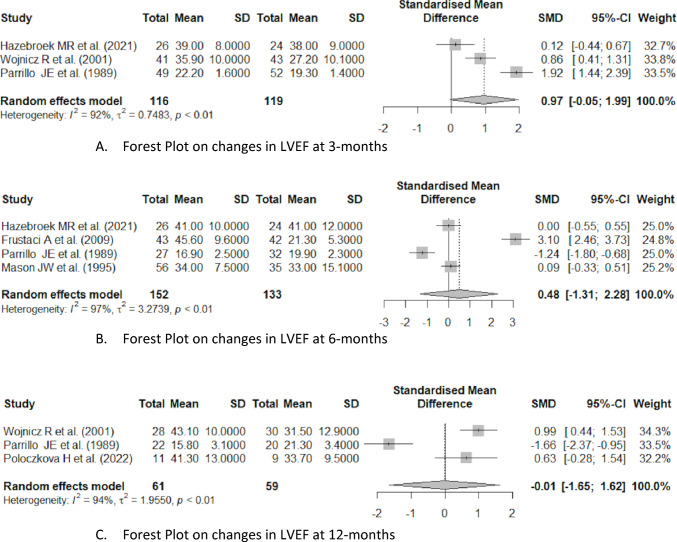
Forest plot on changes in LVEF at 3 months, 6 months and 12 months.

Heterogeneity was substantial (I^2^ = 92%, *p* < 0.01), with *τ*^2^ = 0.748 suggesting considerable between-study variance. This high heterogeneity warrants caution, as the large effect in Parrillo et al.^18^ may be disproportionately influencing the pooled result. The overall pooled SMD from the random effects model was 0.97 [95% CI –0.05, 1.99], suggesting a moderate effect, though the confidence interval crosses zero, indicating no benefit of immunosuppressive treatment (Fig. 2).

At the 6 month follow-up, the effect of immunosuppressive therapy on LVEF was assessed across studies by Hazebroek et al.^13^, Frustaci et al.^16^, Mason et al.^19^, and Parrillo et al.^18^.

Heterogeneity was substantial (I^2^ = 97%, *p* < 0.01), with τ^2^ = 3.274. The overall pooled SMD from the random effects model was 0.48 (95% CI [–1.31, 2.28]), indicating no beneficial effect of immunosuppressive therapy on LVEF at 6 months (Fig. 2).

The 12-month follow-up results on the efficacy of immunosuppression on LVEF are reported by Wojnicz et al.^15^, Parrillo et al.^18^, and Poloczkova et al.^14^.

High heterogeneity (I^2^ = 94%, *p* < 0.01; τ^2^ = 1.955) suggests substantial variability among studies, likely explaining the lack of a statistically significant overall effect.

The overall pooled SMD is –0.01 (95% CI [–1.65, 1.62]), indicating no benefit of immunosuppressive treatment on LVEF at 12 months (Fig. 2).

#### Effects of immunosuppressive therapy related to New York Heart Association Classification (NYHA)

**Fig. 3:**
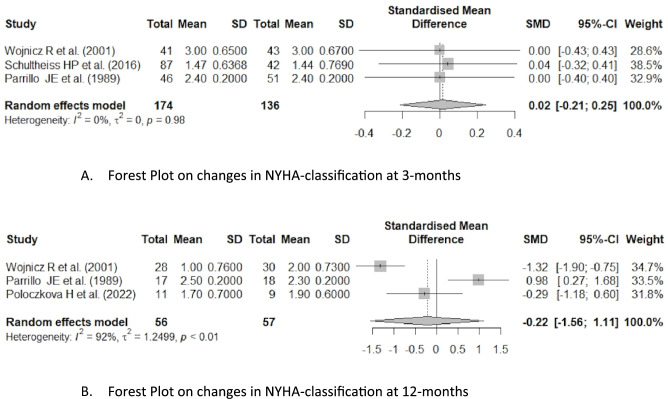
Forest plot on changes in NYHA-classification at 3 months and 12 months.

At the 3 month follow-up, the effect of immunosuppressive therapy on NYHA classification was evaluated in studies by Wojnicz et al.^15^, Schultheiss et al.^17^, and Parrillo et al.^18^. Heterogeneity was absent (I^2^ = 0%, *p* = 0.98, τ^2^ = 0), suggesting consistent findings across studies. However, none of the individual or pooled results showed a beneficial effect of immunosuppressive therapy on the heart failure-specific outcome of NYHA in myocardial inflammation, as all confidence intervals crossed zero. The overall pooled SMD was 0.02 (95% CI [–0.21, 0.25]), indicating no clinical benefit of immunosuppression on NYHA at 3 months. Study weights ranged from 28.6 to 38.5% (Fig. 3).

At the 12 month follow-up, the effect of immunosuppressive therapy on NYHA classification was evaluated in studies by Wojnicz et al.^15^, Parrillo et al.^18^, and Poloczkova et al.^14^. Heterogeneity was substantial (I^2^ = 92%, *p* < 0.01; τ^2^ = 1.25), reflecting considerable variability among studies. These inconsistencies suggest that the treatment effect may vary significantly depending on the specific study or population, warranting caution in interpreting the pooled result. The overall pooled SMD was –0.22 (95% CI [–1.56, 1.11]), indicating no benefit on heart-failure related outcome of NYHA in myocardial inflammation with the use of immunosuppressive therapy (Fig. 3).

## Discussion

Our systematic review and meta-analysis focuses on heart failure-specific outcomes, particularly LVEF and NYHA classification, as these metrics are most often used in clinical practice to guide subsequent medical therapy.

We demonstrated that the use of immunosuppressive therapy in patients with biopsy-proven myocardial inflammation exhibits significant variability in its impact on these heart failure-related outcome measures. While some individual studies report moderate improvements, the overall outcomes remain neutral due to marked inconsistency across trials and substantial heterogeneity. To date, no consistent clinical benefit has been proven for routine use of immunosuppressive therapy in this setting.

A common assumption in clinical practice is that combining immunosuppression with guideline-directed heart failure therapies, such as ACE inhibitors, may promote myocardial recovery in post-inflammatory cardiomyopathy. However, much of the evidence supporting this stems from post-infarction populations, and not from patients with myocardial inflammatory disease. Furthermore, the available data do not support a generalized benefit of immunosuppressive therapy in post-inflammatory heart failure. Exceptions may exist in certain well-defined, often fulminant cases, such as those with hemodynamic compromise, low blood pressure, or cardiogenic shock, where clinicians often act intuitively to initiate immunosuppressive treatment. Nonetheless, these cases represent a minority and should not be generalized to the broader myocarditis population.

Inflammatory myocardial disease can present as acute, chronic, or subacute forms, each with distinct clinical features. Our meta-analysis and systematic review focuses on these prevalent forms to create a consistent patient cohort, deliberately excluding other types such as eosinophilic, giant-cell and fulminant myocarditis, to isolate the effects of treatment across primary inflammatory subtypes. In our review, we identified four previous meta-analyses that explored the role of immunosuppressive therapy in myocarditis or cardiomyopathy resulting from myocarditis^20–23^. These analyses included studies published up to November 2019, leaving two recent randomized controlled trials that have not yet been evaluated in prior reviews, which are assessed and presented in our analysis^13,14^. Moreover, we are the first to assess the clinical benefits of immunosuppressive therapy on heart-failure related outcome measures in patients with biopsy-proven myocardial inflammation and to systematically assess changes in the New York Heart Association (NYHA) classification within a meta-analysis framework, rather than limiting its evaluation to individual study outcomes.

Yet, the results of this meta-analysis indicate a lack of clear evidence for interventions beyond observation and supportive care in most cases. It is important to note that this analysis does not include forms of myocarditis where immunosuppression is well-established, such as eosinophilic myocarditis, giant-cell myocarditis or cardiac sarcoidosis^9,10^.

Despite including these new studies, the results remain neutral, showing no substantial benefit from routine interventions. Furthermore, there is still no evidence to support the routine use of endomyocardial biopsy (EMB) in cases of mild inflammatory myocardial disease, as it does not lead to new therapeutic approaches, even when inflammation is detected.

Biopsy-based inclusion in this meta-analysis serves as both a strength and a limitation. It strengthens internal validity by ensuring that all included patients had histologically confirmed myocardial inflammation, thereby reducing diagnostic uncertainty and enabling a more uniform assessment of immunosuppressive therapy effects. However, this criterion also limits the generalizability of our findings to broader patient populations. Many real-world cases of myocarditis are managed based on clinical and imaging features without biopsy confirmation. As such, our results may not fully apply to patients with milder disease or those in whom biopsy is not feasible or routinely performed. This selection bias may overlook patients who could potentially benefit from therapy but are excluded from trials and clinical decision-making due to lack of biopsy confirmation.

Our pooled analysis of LVEF outcomes showed no clinical benefit of immunosuppressive therapy on this heart failure-related outcome measure in the study population. At the 3-month follow-up, the overall pooled SMD using the random effects model was 0.97, indicating a moderate trend toward favoring immunosuppression. This trend was not sustained at the 6-month and 12 month follow-ups. From a clinical standpoint, our findings do not support a meaningful or sustained improvement in left ventricular systolic function with immunosuppressive therapy in patients with biopsy-proven myocardial inflammation.

Similarly, our pooled analysis of NYHA outcomes did not demonstrate a clinical benefit of immunosuppressive therapy at either the 3 month or 12 month follow-ups.

Heterogeneity remained high throughout our analysis across different time points for both LVEF and NYHA, warranting caution when interpreting the results. The findings from our meta-analysis are hypothesis-generating and should provide an up-to-date overview of the current research on immunosuppressive therapy in myocardial inflammatory disease, as well as generate implications for future studies.

When observing the major inclusion criteria, we noted differences among the included studies. For example, three studies^13,16,19^ used LVEF < 45% as an inclusion criteria, while three other studies^14,15,18^ used an LVEF range of 35–40% as inclusion criteria. Most clinical trials included cases of chronic heart failure^13,15–17^, while Poloczkova et al.^14^ included both acute and chronic cases. Mason et al.^19^ and Parrillo et al.^18^ did not specify, highlighting variability among studies.

The included studies also varied in terms of viral presence and inflammation. Six studies^13–17,19^ included patients with myocardial inflammation, while two studies^13,17^ included patients with viral presence. In addition to this, treatment differences may have contributed to the observed heterogeneity.

Myocardial inflammation is often induced by cardiotropic RNA viruses, such as enteroviruses. However, DNA viruses like Parvovirus B19 (B19V) and human herpesvirus-6 (HHV6) are also frequently detected in endomyocardial biopsies (EMB) of myocarditis patients^1,24^.

The use of immunosuppressive therapy in patients with active viral presence is not endorsed by the European Society of Cardiology (ESC), as it may trigger viral reactivation and replication^25^. Conversely, Tschöpe et al. demonstrated that low levels of B19V DNA do not necessarily preclude the safe and effective use of immunosuppressive therapy under specific conditions^26^. Identifying patient cohorts who may benefit from this therapy requires a thorough evaluation of immune-histochemical and molecular biological analysis of EMBs, as well as autoantibody serum testing^2–5,27–30^. However, the standard use of EMB is limited^2–4^.

Apart from viral presence, chronic myocardial inflammation serves as a predictor of poor outcomes, where early treatment initiation can be lifesaving^4^. Therefore, our meta-analysis aimed to evaluate the effect of immunosuppression on heart failure-related outcome measures, as untreated patients with persistent cardiac inflammation have a higher risk of unfavorable outcomes^31^.

The studies included in our analysis used various treatment regimens. The interventions ranged from IVIG, Prednisone + Azathioprine, Prednisone monotherapy, Interferon-β-1b, to combinations of Azathioprine or Cyclosporine + Prednisone with different treatment durations. This heterogeneity complicates direct comparisons and contributes to variability across studies.

We identified four previous meta-analyses on this topic^20–23^. Timmermanns et al.^20^ and Winter et al.^21^ reported findings consistent with our results. Timmermanns et al.^20^ assessed LVEF as a primary endpoint, as well as mortality and heart transplantation-free survival, using a random effects model like ours. Their analysis, which included registry data with propensity-matched controls and used Azathioprine + Prednisone as the uniform intervention, showed similar outcomes. However, the diagnosis of inflammatory cardiomyopathy was not required to be confirmed through endomyocardial biopsies.

Winter et al.^21^ evaluated LVEF and NYHA, and additionally analyzed EMBs to assess viral clearance and resolution of inflammation. Although they used different interventions, their results suggested a benefit of immunosuppression, but no statistical significance was achieved. However, Lu et al.^22^ presented findings inconsistent with ours, favoring immunosuppressive therapy in myocarditis. Cheng et al.^23^ presented a systematic review and meta-analysis assessing the effect of immunosuppressive therapy on biopsy-proven, virus-negative myocarditis, focusing on primary endpoints such as survival, left ventricular ejection fraction (LVEF), and New York Heart Association (NYHA) classification. However, their analysis included not only patients with lymphocytic myocarditis but also included other types such as eosinophilic and giant-cell myocarditis. They concluded that their results suggest clinical benefits across all primary endpoints. Heterogeneity was a significant challenge in all the mentioned meta-analyses and systematic reviews.

In contrast to previous studies, our systematic review and meta-analysis was able to include two novel randomized clinical trials that have not been analyzed in earlier reviews^13,14^. We focused primarily on heart failure-related outcome measures, with special emphasis on LVEF and NYHA classification. Unlike previous analyses, which assessed NYHA outcomes only in individual studies, we were able to include NYHA in the meta-analysis and generate a pooled result. This provides a more comprehensive assessment of heart failure severity and treatment response across the included trials.

In our analysis, the heart failure-related outcome measures of LVEF and NYHA were considered crucial. Not only were they assessed in most randomized controlled trials, but they also play essential roles in the initiation of therapy and prognosis of heart failure patients. These measures are instrumental in guiding treatment decisions, including the selection of medications and the consideration of implantable devices. LVEF provides a quantitative measure of the heart’s mechanical function, while the NYHA classification offers a subjective, patient-centered assessment. NYHA highlights the impact of heart failure on daily life and functional capacity, offering a more holistic understanding of the patient’s experience.

Together, these two outcome measures enable us to group patients both quantitatively and qualitatively, helping to determine which specific therapies are most appropriate for each patient.

Unlike other reviews, we did not focus on mortality or deaths as endpoint. While myocarditis can lead to severe outcomes in select cases, short-term mortality remains relatively low in non-fulminant presentations. In these patients, clinical focus has shifted toward identifying and managing subtle forms of cardiac injury, with the aim of preventing or slowing disease progression rather than responding to acute deterioration^32^. Thus, tracking functional improvements and symptom relief offers a more informative measure of therapeutic impact. The primary goal in treating myocardial inflammation is to stabilize or improve cardiac function and manage symptoms to prevent progression. LVEF and NYHA provide sensitive markers of these changes, offering a more nuanced assessment of treatment efficacy than mortality alone^33^. Additionally, immunosuppressive therapy may influence cardiac function and symptom burden without directly affecting mortality risk in the short to medium term, making functional outcomes more relevant for evaluating its effectiveness^34^.

Despite applying strict inclusion criteria to enhance methodological rigor, important limitations across the included studies must be acknowledged. There was considerable variability in patient selection, disease chronicity, viral status, endpoint definitions, and immunosuppressive regimens. Not all studies performed viral testing, and treatment durations ranged widely, from just 4 days (Hazebroek et al.^13^) to 6 months (Frustaci et al.^16^, Poloczkova et al. ^14^, Mason et al.^19^) complicating cross-study comparisons.

Mechanistic differences may partially explain the observed heterogeneity. Virus-negative, inflammation-positive patients, as in Frustaci et al.^16^ and Poloczkova et al.^14^ are generally more responsive to immunosuppressive therapy, while virus-positive cases, such as Schultheiss et al.^17^, may benefit less due to persistent pathogen-driven inflammation. The timing of therapeutic intervention is a critical determinant of treatment efficacy, as chronic inflammatory cardiomyopathy may be less responsive to reversal of pathological remodeling than subacute or early-stage disease.

Hazebroek et al.^13^ used IVIG for only 4 days, which may appear short compared to other regimens. However, IVIG operates through distinct immunomodulatory mechanisms and in both pediatric and adult inflammatory cardiomyopathies. Its short-course nature reduces cumulative toxicity risk, while still effectively attenuating acute immune responses that contribute to heart failure progression. These pharmacological properties justify its inclusion and comparability with longer immunosuppressive strategies.

We did not perform subgroup or sensitivity analyses for acute or chronic forms of myocarditis or inflammatory dilated cardiomyopathy. While we recognize the value of such analyses, they were not feasible in our case. The patient groups in the available studies were usually very small, and further subdivision would have compromised the statistical credibility of any subgroup analysis. Another important consideration is that a definitive diagnosis at a single time point is inherently difficult, as these conditions overlap and often evolve from one stage to another, for example from acute to chronic myocarditis and ultimately to inflammatory dilated cardiomyopathy. For this reason, we used inflammation as a unifying denominator across studies, which we consider not a random choice but a thoughtful approach reflecting the biological continuum of these conditions. The boundaries between disease phases are difficult to establish with precision, and our decision to apply this broader conceptual framework further supports the rationale of our meta-analysis.

Overall, the observed variability in immune targets, chronicity, viral status, drug mechanisms, and timing of intervention likely contributes to inconsistent treatment responses. Rather than reflecting a limitation of the analysis itself, this underscores the biological complexity of inflammatory myocardial disease and highlights the need for more precisely targeted therapeutic approaches in future trials.

The key limitation of this meta-analysis is the substantial heterogeneity observed across primary outcomes of LVEF and NYHA. In these analyses, I^2^ values consistently exceeded 90%, indicating considerable heterogeneity as defined by the Cochrane Handbook for Systematic Reviews of Interventions^35^. However, the interpretation of this statistic depends on the magnitude and direction of effects, as well as consistency across studies. In our analysis, the direction of effects remained largely consistent despite variability in effect size. The observed heterogeneity likely reflects clinical and methodological differences across studies, including variations in patient populations, intervention protocols, and follow-up durations, which are common in complex interventions. Importantly, sensitivity analyses confirmed the robustness of the pooled estimates, suggesting that the overall conclusions remain reliable despite this heterogeneity.

In line with PRISMA and Cochrane recommendations, meta-analyses should ideally include an assessment of publication bias using tools such as funnel plots, Egger’s regression test, or Begg’s test. Due to the limited number of studies included in this analysis, we did not perform these tests, as their validity is compromised when fewer than ten studies are available. However, the absence of a formal publication bias assessment should be acknowledged as a methodological limitation.

### Implications for practice, policy, and future research

Given the high heterogeneity in our meta-analysis, there is currently no basis to recommend changes in clinical practice for treating myocardial inflammation. Further research in the form of large, multicenter randomized controlled trials is needed to establish a consensus on whether immunosuppressive therapy should be part of standard care alongside optimal medical therapy. Future trials should standardize methodologies, focusing on similar patient cohorts with a uniform definition of myocardial inflammatory disease. Trials should also employ double-blind designs to reduce performance, detection, and expectation biases associated with open-label studies. Incorporating the use of endomyocardial biopsies more frequently, as they remain the gold standard for diagnosing myocardial inflammation, would improve the understanding of viral latency and viral bystanders and help identify patients who might benefit from immunosuppressive therapy^2–5,27–30^.

We recommend including the most studied interventions, such as Azathioprine + Prednisone, used in previous clinical trials to enhance the validity and understanding of the impact of immunosuppressive interventions on outcomes in patients with myocardial inflammation.

## Supplementary Information

Supplementary Information 1.

Supplementary Information 2.

## Data Availability

All data generated or analyzed during this systematic review and meta-analysis are included in the published article.
