# Supplementary material for: Immunosuppressive therapy in patients with biopsy-proven inflammatory myocardial disease: a systematic review and meta-analysis
Source: Sci Rep. 2025 Oct 23;15:37173. doi: 10.1038/s41598-025-25165-3 (PMC12550003; doi:10.1038/s41598-025-25165-3)
Supplement: Supplementary file 1 — Supplementary Information 1. [file 41598_2025_25165_MOESM1_ESM.docx]

Full electronic search used for PubMed:

Pubmed: Search used: *(((("cardiomyopathy, dilated"[MeSH Terms] OR ("cardiomyopathy"[All Fields] AND "dilated"[All Fields]) OR "dilated cardiomyopathy"[All Fields] OR "cardiomyopathy dilated"[All Fields]) AND "drug therapy*"[MeSH Terms]) OR (("myocardic"[All Fields] OR "myocarditis"[MeSH Terms] OR "myocarditis"[All Fields] OR "myocarditides"[All Fields]) AND "therapy*"[MeSH Terms]) OR (("myocardic"[All Fields] OR "myocarditis"[MeSH Terms] OR "myocarditis"[All Fields] OR "myocarditides"[All Fields]) AND "drug therapy*"[MeSH Terms]) OR "myocarditis*"[MeSH Terms]) AND (("clinical trial"[Publication Type] OR "randomized controlled trial"[Publication Type]) AND 1987/01/01:2022/12/31[Date - Publication])) AND ((clinicaltrial[Filter] OR randomizedcontrolledtrial[Filter]) AND (1989:2022[pdat]))*
