# Supplementary material for: Immunosuppressive therapy in patients with biopsy-proven inflammatory myocardial disease: a systematic review and meta-analysis
Source: Sci Rep. 2025 Oct 23;15:37173. doi: 10.1038/s41598-025-25165-3 (PMC12550003; doi:10.1038/s41598-025-25165-3)
Supplement: Supplementary file 2 — Supplementary Information 2. [file 41598_2025_25165_MOESM2_ESM.docx]

**The Cochrane Collaboration’s tool for assessing risk of bias in randomized trials.** The Risk of Bias 2 (RoB 2) tool assesses the risk of bias in randomized trials, assessing (1) Randomization Process, (2) Deviations from the intended interventions, (3) Missing outcome data, (4) Measurement of the outcome, (5) Selection of the reported result, (6) Overall risk of bias

**Legend:** NI = no information, PY = probably yes, Y = Yes, PN = probably no, N = no

**Overall risk-of-bias judgement Criteria**

**Low risk of bias**: The study is judged to be at low risk of bias for all domains for this result.

**Some concerns**: The study is judged to raise some concerns in at least one domain for this result, but not to be at high risk of bias for any domain.

**High risk of bias**: The study is judged to be at high risk of bias in at least one domain for this result.

Or

The study is judged to have some concerns for multiple domains in a way that substantially lowers confidence in the

result.

| Study | Hazebroek - 2021 | Wojnicz – 2001 | Frustaci – 2009 | Schultheiss- 2016 | Parrillo - 1989 | Poloczkova – 2022 | Mason - 1995 |
| --- | --- | --- | --- | --- | --- | --- | --- |
| 1.1 | NI | NI | NI | NI | NI | NI | NI |
| 1.2 | Y | NI | Y | PY | NI | NI | NI |
| 1.3 | N | N | N | PN | PN | PN | PN |
| **1.0 Algorithm result** | Low | Some concerns | Low | Low | Some concerns | Some concerns | Some concerns |
| **1.0 Assessor‘s judgement** | Low | Some concerns | Low | Low | Some concerns | Some concerns | Some concerns |
| 2.1 | N | NI | PN | N | Y | Y | PY |
| 2.2 | N | NI | N | PN | Y | Y | Y |
| 2.3 | NA | PN | NA | NA | PN | PN | PN |
| 2.4 | NA | NA | NA | NA | NA | NA | NA |
| 2.5 | NA | NA | NA | NA | NA | NA | NA |
| 2.6 | PY | Y | PY | PY | PN | NI | PY |
| 2.7 | NA | NA | NA | NA | PN | PN | NA |
| **2.0 Algorithm result** | Low | Low | Low | Low | Some concerns | Some concerns | Low |
| **2.0 Assessor’s judgement** | Low | Low | Low | Low | Some concerns | Some concerns | Low |
| 3.1 | Y | PN | Y | PY | PN | PY | PN |
| 3.2 | NA | PN | NA | NA | PN | NA | PY |
| 3.3 | NA | PN | NA | NA | PY | NA | NA |
| 3.4 | NA | NA | NA | NA | NI | NA | NA |
| **3.0 Algorithm result** | Low | Low | Low | Low | High | Low | Low |
| **3.0 Assessor’s judgement** | Low | Low | Low | Low | High | Low | Low |
| 4.1 | N | N | N | N | N | N | N |
| 4.2 | N | N | N | PN | N | N | N |
| 4.3 | N | PY | N | PN | Y | Y | NI |
| 4.4 | NA | PY | NA | NA | PN | PY | PY |
| 4.5 | NA | PN | NA | NA | NA | PN | PN |
| **4.0 Algorithm result** | Low | Some concerns | Low | Low | Low | Some concerns | Some concerns |
| **4.0 Assessor’s judgement** | Low | Some concerns | Low | Low | Low | Some concerns | Some concerns |
| 5.1 | Y | NI | PY | PY | NI | PY | NI |
| 5.2 | N | N | N | N | NI | PN | N |
| 5.3 | N | N | N | N | NI | PN | N |
| **5.0 Algorithm result** | Low | Some concerns | Low | Low | Some concerns | Low | Some concerns |
| **5.0 Assessor’s judgement** | Low | Some concerns | Low | Low | Some concerns | Low | Some concerns |
| *Algorithm’s overall judgement* | Low | Some concerns | Low | Low | High | Some concerns | Some concerns |
| *Assessor’s overall judgement* | Low | Some concerns | Low | Low | High | Some concerns | Some concerns |

**Y; Yes, PY; probably yes, PN; probably no, N; No, NI; No information**

1.1 Was the allocation

sequence random?

1.2 Was the allocation

sequence concealed until

participants were

enrolled and assigned to

interventions?

1.3 Did baseline

differences between

intervention groups

suggest a problem with

the randomization

process?

2.1. Were participants

aware of their assigned

intervention during the

trial?

2.2. Were carers and

people delivering the

interventions aware of

participants' assigned

intervention during the

trial?

2.3. If Y/PY/NI to 2.1 or

2.2: Were there

deviations from the

intended intervention

that arose because of the

trial context?

2.4 If Y/PY to 2.3: Were

these deviations likely to

have affected the

outcome?

2.5. If Y/PY/NI to 2.4:

Were these deviations

from intended

intervention balanced

between groups?

2.6 Was an appropriate

analysis used to estimate

the effect of assignment

to intervention?

2.7 If N/PN/NI to 2.6:

Was there potential for a

substantial impact (on

the result) of the failure

to analyse participants in

the group to which they

were randomized?

3.1 Were data for this

outcome available for all,

or nearly all, participants

randomized?

3.2 If N/PN/NI to 3.1: Is

there evidence that the

result was not biased by

missing outcome data?

3.3 If N/PN to 3.2: Could

missingness in the

outcome depend on its

true value?

3.4 If Y/PY/NI to 3.3: Is it

likely that missingness in

the outcome depended on

its true value?

4.1 Was the method of

measuring the outcome

inappropriate?

4.2 Could measurement

or ascertainment of the

outcome have differed

between intervention

groups?

4.3 If N/PN/NI to 4.1 and

4.2: Were outcome

assessors aware of the

intervention received by

study participants?

4.4 If Y/PY/NI to 4.3:

Could assessment of the

outcome have been

influenced by knowledge

of intervention received?

4.5 If Y/PY/NI to 4.4: Is it

likely that assessment of

the outcome was

influenced by knowledge

of intervention received?

5.1 Were the data that

produced this result

analysed in accordance with

a pre-specified analysis plan

that was finalized before

unblinded outcome data

were available for analysis?

5.2. ... multiple eligible

outcome measurements

(e.g. scales, definitions,

time points) within the

outcome domain?

5.3 ... multiple eligible

analyses of the data?
